# Functional deterioration of vascular mitochondrial and glycolytic capacity in the aortic rings of aged mice

**DOI:** 10.1007/s11357-024-01091-6

**Published:** 2024-02-28

**Authors:** Agnieszka Karaś, Anna Bar, Kanchana Pandian, Agnieszka Jasztal, Zuzanna Kuryłowicz, Barbara Kutryb-Zając, Elżbieta Buczek, Stefano Rocchetti, Tasnim Mohaissen, Agata Jędrzejewska, Amy C. Harms, Patrycja Kaczara, Stefan Chłopicki

**Affiliations:** 1 Jagiellonian Centre for Experimental Therapeutics, Jagiellonian University, Bobrzynskiego 14, 30-348, Krakow, Poland; 2 Doctoral School of Exact and Natural Sciences, Jagiellonian University, Lojasiewicza 11, 30-348, Krakow, Poland; 3 Leiden Academic Centre for Drug Research, Leiden University, Einstein Road 55, 2333 CC Leiden, The Netherlands; 4 Department of Biochemistry, Medical University of Gdansk, Debniki 1, 80-211, Gdansk, Poland; 5 Department of Pharmacology, Jagiellonian University Medical College, Grzegorzecka 16, 31-531, Krakow, Poland

**Keywords:** Mitochondrial function, Glycolysis, Aorta, Ageing, NAD, Vascular stiffness, Seahorse XF technique

## Abstract

Vascular ageing is associated with increased arterial stiffness and cardiovascular mortality that might be linked to altered vascular energy metabolism. The aim of this study was to establish a Seahorse XFe96 Analyzer-based methodology for the reliable, functional assessment of mitochondrial respiration and glycolysis in single murine aortic rings and to validate this functional assay by characterising alterations in vascular energy metabolism in aged mice. Healthy young and old C57BL/6 mice were used for the analyses. An optimised setup consisting of the Seahorse XFe96 Analyzer and Seahorse Spheroid Microplates was applied for the mitochondrial stress test and the glycolysis stress test on the isolated murine aortic rings, supplemented with analysis of NAD content in the aorta. To confirm the age-dependent stiffness of the vasculature, pulse wave velocity was measured in vivo. In addition, the activity of vascular nitric oxide synthase and vascular wall morphology were analysed ex vivo. The vascular ageing phenotype in old mice was confirmed by increased aortic stiffness, vascular wall remodelling, and nitric oxide synthase activity impairment. The rings of the aorta taken from old mice showed changes in vascular energy metabolism, including impaired spare respiratory capacity, maximal respiration, glycolysis, and glycolytic capacity, as well as a fall in the NAD pool. In conclusion, optimised Seahorse XFe96-based analysis to study energy metabolism in single aortic rings of murine aorta revealed a robust impairment of functional vascular respiratory and glycolytic capacity in old mice linked to NAD deficiency that coincided with age-related aortic wall remodelling and stiffness.

**Supplementary Information:**

The online version contains supplementary material available at 10.1007/s11357-024-01091-6.

## Introduction

Vascular ageing is a key risk factor for cardiovascular diseases. Two specific phenotypic features related to age, systemic endothelial dysfunction, and stiffness of the large arteries predispose individuals to cardiovascular morbidity and mortality [1]. Although the vascular ageing phenotype can occur even in young people [2], the increase in life expectancy worldwide has resulted in a significant increase in the incidence of age-related cardiovascular disease [3].

Several studies have shown that vascular ageing is associated with mitochondrial dysfunction [4], which contributes to increased aortic stiffness [5, 6] and the development of atherosclerosis, particularly in aged mice [7]. Mitochondrial dysfunction was also suggested to participate in the development of age-dependent endothelial dysfunction [8], a notion supported by a recent report showing that mitochondrial ATP production maintained endothelium-dependent modulation of vascular tone [9], despite the documented dominant role of glycolysis in endothelial cells [10–12].

Furthermore, vascular ageing was associated with a decrease in NAD levels [13], the secretion of pro-inflammatory factors [13], increased vascular oxidative stress [14], and DNA damage [15], all of which could alter mitochondrial energy metabolism. Vascular ageing was also shown to be associated with impaired mitochondrial quality control, whilst the stimulation of mitophagy can reduce age-dependent aortic stiffening [6]. Interestingly, the induction of mitochondrial stress by rotenone led to increased aortic stiffness in young mice [6]. On the other hand, therapies targeted to vascular metabolism that aim to restore NAD levels, including precursors of NAD, improved various age-related vascular phenotypes [16–18].

Collectively, these studies outline the critical role of impaired mitochondrial function in age-dependent deterioration of the vascular phenotype. However, the insight into the functional aspect of vascular energy metabolism in murine conduit vessels, such as the aorta, is limited mainly to studies on cultured primary cells and isolated mitochondria, supplemented with fluorescence imaging of mitochondria-targeted dyes in en face aortic preparation. Due to methodological constraints, studies on vascular energy metabolism are not frequently carried out in the preparation of isolated vessels, although such a model takes into the measurement scenario a metabolite-based crosstalk between various cell types in the vascular wall and could provide additional insight. To date, only a few attempts have been made to measure vascular energy metabolism directly in isolated murine vessels ex vivo [7, 19]*.* The methods previously used were not suitable for single murine aortic rings, either requiring a whole aorta [7] or larger parts of aortic sections [19], thus minimizing the number of readouts from a single murine aorta and not allowing researchers to expose fragments of the aorta from the same animal to different experimental conditions. Hence, there is still an emerging need for a methodology that allows reproducible and high-quality measurements of glycolytic and mitochondrial function in the single aortic rings in order to obtain a reliable insight into vascular energy metabolism in the murine aorta ex vivo and at the same time to allow multiple comparisons with technical and biological replicates in the same assay.

Therefore, the purpose of our work was to optimise the Seahorse XFe96 technique with the application of Seahorse Spheroid Microplates to measure mitochondrial respiration and glycolysis in minute murine aortic rings and to verify whether this experimental approach could detect deterioration of the functional profile of vascular energy metabolism in aged mice. We demonstrated that vascular ageing reflected by aortic wall remodelling and increased stiffness in vivo was accompanied by functional impairment of mitochondrial and glycolytic reserve in the vascular wall, fully confirming the robustness of this methodology.

## Materials and methods

### Animal models

Studies were carried out on C57BL/6 male mice from the Mossakowski Medical Research Centre, Polish Academy of Sciences, Warsaw, Poland, or Warsaw Medical University, Warsaw, Poland. Age-related comparisons were performed using young (3–4 months), adult (8–10 months), and old (22–26 months old) C57BL/6 mice. The mice were housed under specific pathogen-free conditions (SPF) in a room with constant environmental conditions (22–25 °C, 65–75% humidity, and a 12-h light/dark cycle) and fed with a standard laboratory diet and water ad libitum. The size of a given experimental group is reported in the legends of the corresponding graphs. All experiments were approved by the Ethics Local Committee of Jagiellonian University (Krakow, Poland) and were performed according to the Guide for the Care and Use of Laboratory Animals of the National Academy of Sciences (National Institutes of Health publication 85–23, revised 1996), as well as the Guidelines for Animal Care and Treatment of the European Community. Basic health conditions of the mice were evaluated before the experiments, and there were no exclusions from the studies.

### Analysis of vascular energy metabolism in isolated aortic rings using the Seahorse XFe96 Analyzer

The oxygen consumption rate (OCR), reflecting mitochondrial respiration, and the extracellular acidification rate (ECAR), reflecting glycolysis, were measured in single murine aortic rings ex vivo using the Seahorse XFe96 Analyzer (Agilent, USA). Previously, the Seahorse XF24 Analyzer was applied by Feeley et al. for the analysis of mitochondrial function in approximately 3-mm-wide aortic rings opened longitudinally using the mitochondrial stress test (MST) protocol [19]. We based our method on the method described by Feeley et al., with significant modifications to adapt the protocol to the 96-well system and the spheroid microplates to allow measurements in single aortic rings approximately 1 mm wide. Furthermore, in addition to MST based on sequential additions of oligomycin (10 µg/ml; Sigma-Aldrich, USA), FCCP (carbonyl cyanide 4-[trifluoromethoxy]phenylhydrazone; 1 µM; Sigma-Aldrich, USA), and rotenone (5 µM; Sigma-Aldrich, USA) with antimycin A (5 µM; Sigma-Aldrich, USA), we also performed a glycolysis stress test (GST) based on sequential additions of glucose (10 mM; MERCK, USA), oligomycin (10 µg/ml), and 2-deoxyglucose (2DG; 50 mM; Sigma-Aldrich, USA).

**Fig. 1:**
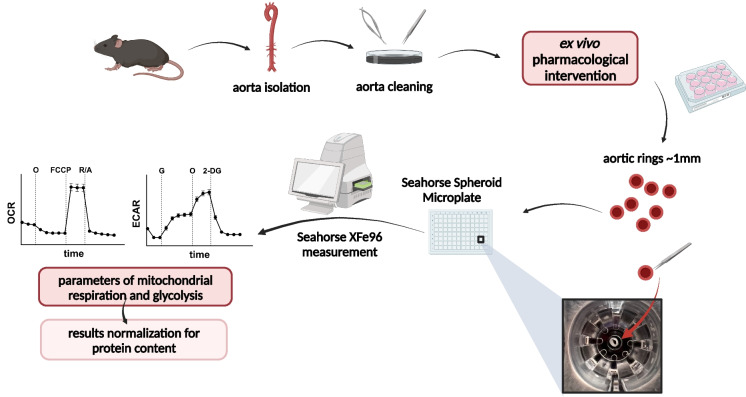
Graphical presentation of the experimental protocol of the measurement of vascular bioenergetics in murine isolated aortic rings using Seahorse XFe96 Analyzer (created with BioRender.com). O – oligomycin, FCCP – carbonyl cyanide 4-[trifluoromethoxy]phenylhydrazone, G – glucose, 2-DG – 2-deoxyglucose

Before the assay, the sensor cartridge was hydrated in Seahorse XF Calibrant solution overnight at 37 °C without CO_2_. The assay media based on Agilent Seahorse XF Base Medium Minimal DMEM (Agilent, USA) and working solutions were prepared freshly on the day of the experiment. For the MST, the assay medium was supplemented with glucose (5.5 mM), glutamine (2 mM; Sigma-Aldrich, USA), and pyruvate (1 mM; Sigma-Aldrich, USA), whilst for the GST, the assay medium was supplemented with glutamine (2 mM) only. The thoracic part of the aorta below the aortic arch was isolated, cleaned of surrounding tissues, and used directly for measurements. Just before measurement, freshly isolated aorta fragments were cut into even rings ~ 1 mm long. The medium was prewarmed at 37 °C on a preheated pad and excessive air bubbles were removed from the plate wells using an optical loupe and a single-channel pipette. The aortic rings were then carefully placed on the Seahorse XFe96 Spheroid Microplate (Agilent, USA) in the assay medium, in the middle of a well with vessel walls perpendicular to the plate bottom (Fig. 1), and incubated for 1 h at 37 °C in the absence of CO_2_ prior to the start of the assay. One aortic ring was placed in each well of the multi-wall plate, and four technical replicates were used for each aorta fragment. Although preliminary experiments were performed with the application of Corning Cell-Tak Cell and Tissue Adhesive (data not shown), the perpendicular arrangement of the aortic rings allowed the tissue glue to be avoided, because the aortic rings remained at the bottom of their wells during the measurements. Importantly, in the Seahorse measurement protocol, the mix–wait–measure times were 3 min–2 min–3 min, with an especially important wait step allowing equilibration of oxygen level in the assay medium. All measurements were normalised to the total protein content (described in detail below). Data were recorded and analysed using Wave software (Agilent). Calculations of energy metabolism parameters were performed according to Agilent MST and GST user guides.

### Measurement of protein concentration in small aortic rings

After the functional metabolic assay, the aortic rings were homogenised to measure protein concentration. Briefly, the aortic rings were transferred to 0.5-ml Precellys Soft Tissue Homogenizing tubes (CK14; Bertin, USA) containing 40 μl of T-PER™ Tissue Protein Extraction Reagent (Thermo Fisher, USA) with the addition of MS-SAFE Protease and Phosphatase Inhibitor (Sigma-Aldrich, USA). Tissue disruption was performed using the Precellys Evolution Tissue Homogeniser (Bertin, USA), 3 times at a speed of 7800 rpm. Then, all samples were incubated for 15 min in an ultrasound bath filled with ice to prevent heating. The homogenised samples were transferred to fresh Protein LoBind® tubes (Eppendorf, Germany) and centrifuged (15 min, 16,602*g*, 4 °C); supernatants were then transferred again to fresh Protein LoBind® tubes. Protein concentration in the aortic homogenates was measured with the Pierce™ BCA protein assay kit (Thermo Fisher, USA) and the total protein content was used to normalise the results of the functional metabolic assay results.

### Measurement of NAD and NADH content in the isolated aorta

Briefly, the isolated thoracic aorta was incubated for 2 h in 37 °C in Krebs-Henselheit buffer, and then the samples were snap-frozen in liquid nitrogen. Tissue extracts were prepared using 0.4 M perchloric acid. After centrifugation (15 min, 13,000*g*, 4 °C) supernatants were neutralised with 3M K_3_PO_4_ and centrifuged again. Analysis of the metabolites was performed with the HPLC method using the LC system (Nexera LC40 set and an SPD-M30A diode array detector equipped with a high-sensitivity, 85-mm optical path cell, Shimadzu, Japan) [20]. As adenosine 5′-diphosphoribose (ADPR) is the major product formed by acidic cleavage of NADH during the tissue extraction, the measured ADPR level reflected NADH concentration in our experimental setting [21, 22].

### Analysis of pulse wave velocity, an index of vascular stiffness in vivo

Aortic pulse wave velocity (PWV) was measured using a Doppler flow velocity system (Indus Instruments, Scintica Instrumentation). Briefly, mice were anaesthetised with 1.5 vol% isoflurane (Aerrane; Baxter Sp. z o. o.) mixed with oxygen. PWV measurements were performed using 20-MHz Doppler probes, by simultaneously recording velocity signals from separate sites, separately for the thoracic and abdominal aorta, as previously described [23].

### Analysis of arginine metabolism in the isolated aorta using tracer-based metabolomics

Tracer-based metabolomics was performed using labelled arginine to evaluate NOS and arginase activity in the aorta based on previously described methodology [24]. Briefly, the isolated thoracic aorta was incubated for 24 h in RPMI SILAC culture medium containing 1% FBS and 150 μM ^13^C_6_, ^15^N_4_ L-arginine-HCl (Sigma-Aldrich, USA); calcium ionophore A23187 (1 µM; Cayman Chemical, USA) was added for the last 90 min of incubation. The effluents were collected and stored at − 80 °C. The preincubation time of aorta with labelled L-arginine was chosen based on preliminary experiments and was set to 24-h period, to ensure that labelled L-arginine is used for NO synthesis to detect labelled L-citrulline derived from nitric oxide synthase (NOS) activation induced by calcium ionophore A23187. For metabolic analysis, the effluent samples (500 µl) were dried under a speed vacuum, followed by reconstitution in 100 µl of Milli-Q water. The samples were analysed using a method based on amine profiling platform that employed an AccQ-TagAQC derivatisation strategy (Waters, Waters B.V. Art. No. 186003836, The Netherlands) adapted from the protocol provided by Waters [25]. Measurements were performed using an ultra-performance liquid chromatography (UPLC) Class I (Acquity, Waters Chromatography Europe BV, Etten-Leur, The Netherlands) system with an AccQ-Tag Ultra C18 Column (1.7 μm, 100 × 2.1 mm, Waters, Ireland) coupled to a Sciex QTRAP® 6500 mass spectrometer as previously described [24].

### Histological assessment of the vascular wall phenotype

Histological assessment of the vascular wall phenotype was performed in 5-µm-thick cross sections from the thoracic aorta below the aortic arch as described elsewhere [26, 27]. Aortic slices were prepared and stained with haematoxylin and eosin (HE) for general morphology and picrosirius red (PSR) to visualise collagen fibres. Slices were scanned with a BX51 microscope equipped with the virtual microscopy system dotSlide (Olympus; Tokyo, Japan). Quantitative analysis of stained images was obtained via image segmentation adopted in Ilastik software. Images captured after PSR staining were used to measure the media thickness as previously described [28]. The collagen fibre content in the aortic media using the image segmentation method was adopted in Ilastik software to calculate the number of red pixels; the results are expressed as a percentage of all pixels in the aortic media area.

### Statistical analyses

Statistical analyses were performed using GraphPad Prism Software v9.5 (GraphPad Software, USA). All results are presented as mean ± SEM. Normality was tested with Shapiro–Wilk test. Comparisons between groups were performed using ANOVA with post hoc Tukey or Bonferroni testing, paired or unpaired *t*-tests as appropriate; significance was considered as a value of *P* ≤ 0.05.

## Results

### Aortic stiffness, vascular wall remodelling, and impaired activity of NOS in vascular wall in old C57BL/6 mice

**Fig. 2:**
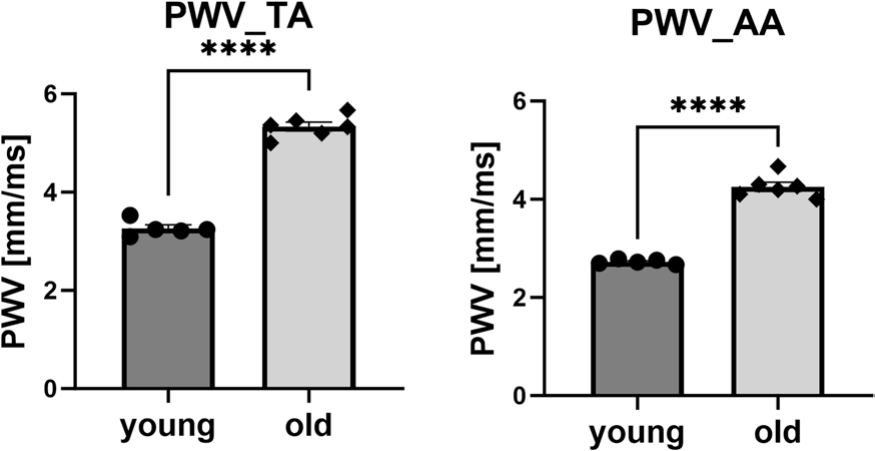
Increased vascular wall stiffness measured in vivo in the aorta of old C57BL/6 mice. Aortic stiffness was assessed as pulse wave velocity (PWV) index in old (24-month-old) as compared to young (3-month-old) C57BL/6 mice in the thoracic (TA) and abdominal (AA) aorta. Data represent the means ± SEM (*n* = 5–6), analysed with *t*-test, ****P ≤ 0.001

Old C57BL/6 mice displayed increased arterial stiffness, as evidenced by an increase in PWV in the thoracic aorta compared with young C57BL/6 mice (Fig. 2). PWV was also increased in the abdominal aorta in old mice compared with young C57BL/6 mice (Fig. 2).

**Fig. 3:**
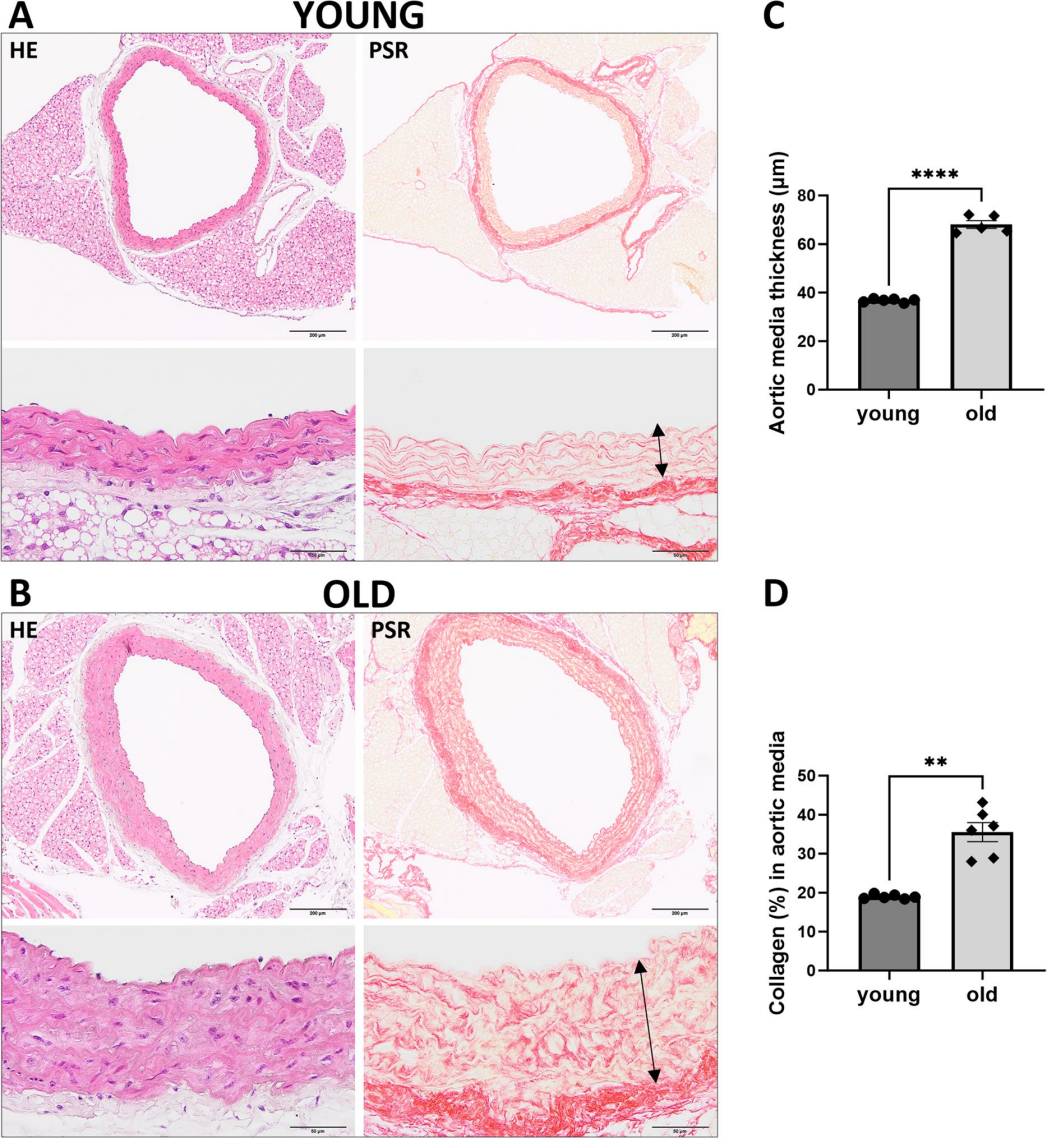
Aortic wall remodelling in old C57BL/6 mice using histological analysis. Representative images of haematoxylin and eosin (HE) staining (*n* = 6) and collagen fibre staining using picrosirius red (PSR) (*n* = 6) in the aorta of old (25-month-old) (**B**) as compared with young (3-month-old) C57BL/6 mice (**A**) in 100 × magnification (top panel) and 400 × magnification (bottom panel). **C** Aortic media thickness measured on PSR staining images, as demonstrated with arrows in the figures A and B. Data represent means ± SEM (*n* = 5–6), analysed with *t*-test, ****P ≤ 0.0001. **D** Relative collagen content in media assessed as a percentage of red pixels on PSR-stained images. Data represent means ± SEM (*n* = 6), analysed with *t*-test, **P ≤ 0.01

Vascular wall histology revealed several changes in the structure of aortic tissue in old C57BL/6 mice as compared with young mice; hypertrophy of the vascular smooth muscle cell (VSMC) layer was visible by haematoxylin and eosin (HE) staining and fibrosis was evidenced by picosirius red (PSR) staining of collagen fibres (Fig. 3A, B). There was a significant increase in media thickness related to smooth muscle cell remodelling (Fig. 3C) and increased collagen content in the vascular wall media in old mice as compared to young (Fig. 3D).

**Fig. 4:**
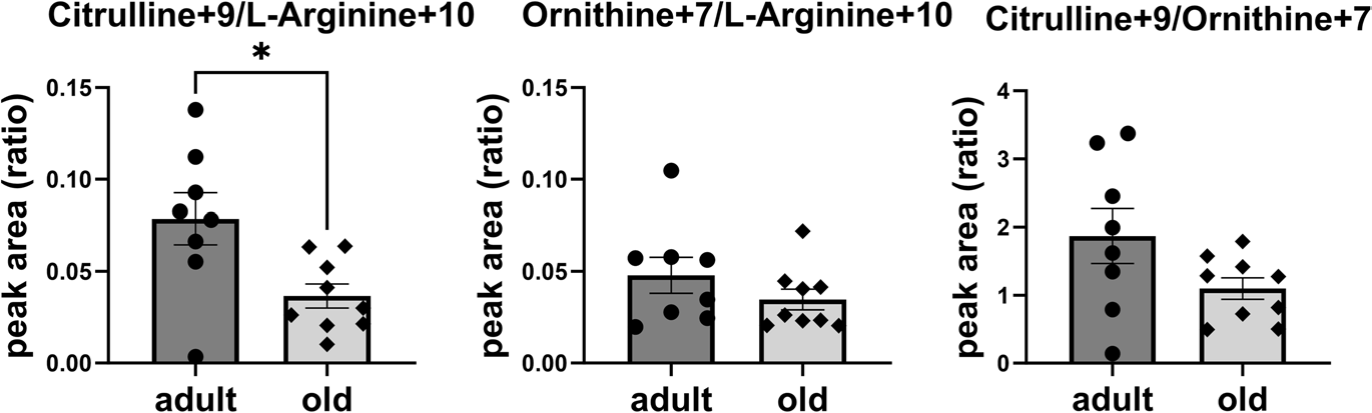
Impaired NOS activity in the aorta of old C57BL/6 mice assessed by the metabolism of stable isotope labelled L-arginine. Aorta isolated from adult (8-month-old) and old (26-month-old) C57BL/6 mice was incubated for 24 h with ^13^C_6_, ^15^N_4_ L-arginine-HCl (arginine + 10) and stimulated with calcium ionophore for the last 90 min. The levels of labelled arginine and its metabolites, ^13^C_6_, ^15^N_3_ L-citrulline (citrulline + 9) and ^13^C_5_, ^15^N_2_ L-ornithine (ornithine + 7) were measured using LC/MS method and expressed as relative ratios. Data represent means ± SEM (*n* = 8–9), analysed with *t*-test/Mann–Whitney, *P ≤ 0.05

Aged C57BL/6 mice also exhibited a decrease in the labelled citrulline level and citrulline/arginine ratio, indicating a decrease in arginine utilisation by NOS for NO production (Figs. 4 and S1). In turn, the labelled ornithine/arginine ratio was not altered, indicating unchanged arginine utilisation by arginase in old mice. The labelled citrulline/ornithine ratio, indicating relative NOS to arginase activity, appeared to be lower in old mice, but the difference did not reach statistical significance. The age-related decline in vascular NO production was confirmed by direct measurements of vascular NO production using electron paramagnetic resonance (EPR) spectroscopy (Figure S2).

### Alterations in the vascular energy metabolism profile in old C57BL/6 mice

**Fig. 5:**
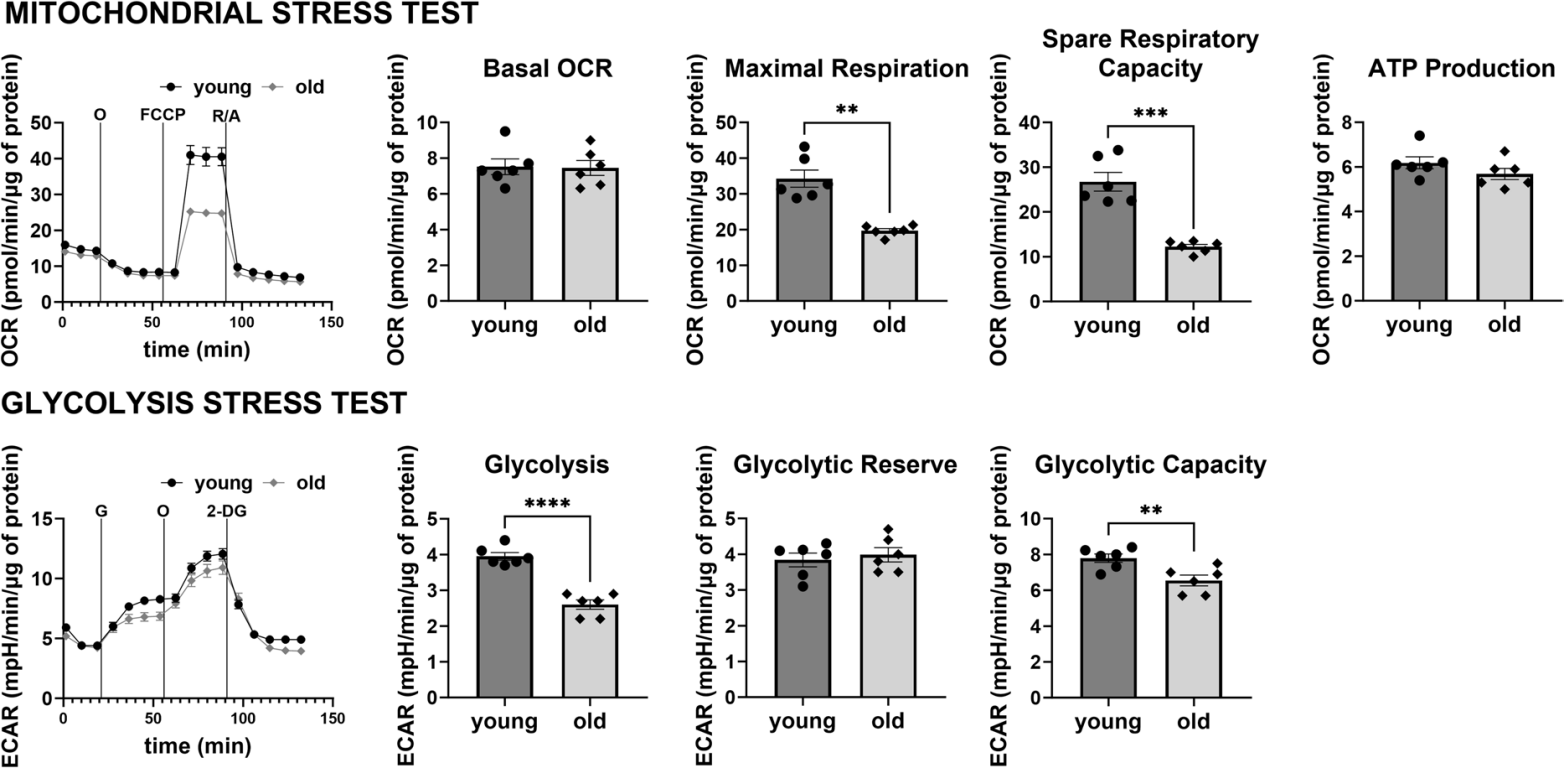
Alterations in functional vascular energy metabolism profile in aorta from old C57BL/6 mice as compared with young C57BL/6 mice measured in single aortic rings using the Seahorse XF Analyzer, with mitochondrial stress test (MST) and the glycolysis stress test (GST). Basal bioenergetics were assessed in aortic rings isolated from old (25-month-old) C57BL/6 male mice compared with young (3-month-old) mice using the Seahorse XFe96 Extracellular Analyzer and mitochondrial stress test protocol and glycolytic stress test. Data represent the means ± SEM (*n* = 6), analysed with *t*-test, *P ≤ 0.05, **P ≤ 0.01, ***P ≤ 0.001, ****P ≤ 0.001

**Fig. 6:**
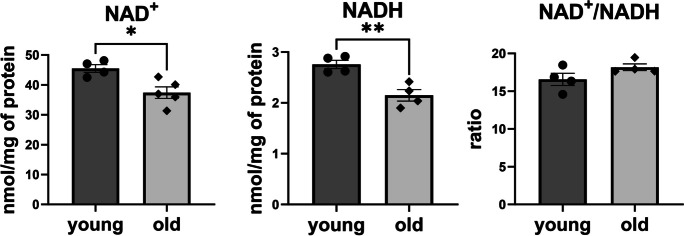
Fall in the content of nicotinamide adenine dinucleotide (NAD) in the aorta of old C57BL/6 mice as compared with young C57BL/6 mice. Nicotinamide adenine dinucleotide concentrations in both oxidised (NAD.^+^) and reduced form (NADH) were assessed using HPLC method in young (4-month-old) and old (25-month-old) C57BL/6 mice in the thoracic aorta. Data represent the means ± SEM (*n* = 4), analysed with *t*-test, *P ≤ 0.05, **P ≤ 0.01

To evaluate the effect of ageing on mitochondrial function and glycolysis in the vascular wall, the mitochondrial stress test (MST) and the glycolysis stress test (GST) were performed in isolated aortic rings of young and old mice. In the aorta of aged mice, a significant decrease in maximal respiration (by 42%) and spare respiratory capacity (by 54%) was observed (Fig. 5). Notably, the basal OCR and ATP production-linked OCR remained unchanged. The aortic rings of old mice also displayed a reduction in basal glycolysis by 34% and glycolytic capacity by 16% (Fig. 5). In adult mice (10 months of age), no changes in energy metabolism parameters in the aorta were observed compared with young mice (Figure S3). Furthermore, old C57BL/6 mice showed a significant decrease in both NAD^+^ and NADH contents in the isolated aorta compared to young mice, whilst the NAD/NADH ratio was unaffected (Fig. 6).

## Discussion

In previous studies, age-related mitochondrial dysfunction was proposed to contribute to aortic stiffness [4, 6], whilst mitochondria-targeted therapies improved vascular health upon ageing [5, 6, 8]. Here, taking advantage of the optimised methodology of functional profiling of vascular energy metabolism in single aortic rings, we expanded this knowledge. Namely, we showed that in the stage of increased age-dependent arterial stiffness (Fig. 2), associated with vascular wall remodelling (Fig. 3), maximal respiration and glycolytic capacity were both impaired (Fig. 5). It clearly indicated impaired vascular energy metabolism reserve in the vascular wall of old mice.

Previously, a metabolic switch from aerobic to anaerobic metabolism was reported in the vascular wall in ageing [29]. In our study, the basal and ATP production-linked levels of mitochondrial respiration were preserved in old mice, but maximal respiration was impaired together with impaired glycolytic capacity (Fig. 5). Importantly, we detected a decrease in the NAD pool in the aorta of aged mice (Fig. 6), which could have reflected mitochondrial dysfunction in ageing [30]. Indeed, since NAD availability is necessary for cellular energy turnover—in an oxidised form (NAD^+^) for glycolysis and in a reduced form (NADH) for mitochondrial respiration—a fall in the vascular NAD pool is likely to explain not only the impaired respiratory capacity but also impaired glycolysis reserve as reported here. These results stay in line with previous report performed on isolated presenescent primary endothelial cells that has shown impairment of glycolysis and glycolytic capacity and decreased mitochondrial respiratory capacity [31]. Furthermore, supplementation with NAD precursors could improve vascular mitochondrial capacity [32] and rescue age-related changes in mitochondrial gene expression in aged mice [33]. NAD precursors were also shown to mitigate age-dependent development of arterial stiffness by normalizing collagen production [34] and have anti-atherogenic effects in the aorta of aged mice [35]. Interestingly, aortic VSMCs isolated from old rats with low aerobic exercise capacity had impaired respiratory capacity compared with cells from old rats with high aerobic exercise capacity [36]. Finally, the protective effects of aerobic exercise in vascular ageing were attributed in part to the enhancement of NAD synthesis [37, 38]. Taken together, our results are consistent with numerous reports suggesting that NAD deficiency contributes to vascular ageing. Accordingly, NAD-targeted therapy represents an interesting therapeutic avenue for the ageing vasculature to orchestrate age-related reprogramming of vascular wall metabolism.

In the present work, we demonstrated that age-dependent aortic stiffness was associated with vascular remodelling, as evidenced by hypertrophy and alterations in the vascular smooth muscle layer of the aortic wall and increased collagen deposition (Fig. 3), a histological picture compatible with stiffening of the large elastic artery [39]. Of note, it was a vascular smooth muscle phenotypic change that included, for example a switch to a synthetic phenotype of VSMCs producing extracellular matrix and collagen that participated in the pathogenesis of various vascular diseases [40] or in inflammageing as a source of pro-inflammatory factors [41]. Such a phenotypic switch of vascular smooth muscle cells could be related to metabolic reprogramming. For example, metabolic reprogramming of VSMCs was suggested to play a critical role in the pathogenesis of thoracic aortic dissection involving mitochondrial ROS damage and altered mTOR signalling [42]. Further studies are needed to understand better the metabolic mechanisms underlying the phenotypic switch in VSMCs that contribute to age-dependent arterial stiffness and remodelling and to uncover the details of the metabolic reprogramming detected here as functional impairment of respiratory and glycolytic reserve associated with NAD deficiency [43–45].

We also demonstrated that age-dependent aortic stiffness was associated with impaired vascular NOS activity (Figs. 4, S1, and S2). These results are consistent with an accepted view that NO-dependent function regulates vascular stiffness [46, 47], whilst pharmacological intervention that improves NO-dependent function results in a reduction in vascular stiffness [48–52]. Impaired vascular bioavailability of NO could contribute to an age-related decrease in spare respiratory capacity by oxidative stress-mediated reduction in the activity of mitochondrial respiratory chain complexes [53, 54]. In fact, the superoxide anion in the reaction with NO forms peroxynitrate [55] that can cause oxidative damage to numerous mitochondrial proteins, for example complexes I and II of ETC or ATP synthase [56], suggesting that mitochondria-targeted antioxidants could prevent impaired vascular energy metabolism in ageing. In fact, the age-related decline in mitochondrial respiratory function and NO-dependent function was reversed in aged individuals by MitoQ, a mitochondria-targeted antioxidant [57].

An important asset of this study was that we have optimised MST and GST assays for single aortic ring measurements using the Seahorse XFe96 analyser, evaluating parameters of both mitochondrial respiration and glycolysis that provided insight into the vascular wall energy metabolism of the murine aorta. Previously, several research groups attempted to analyse isolated vessel energy metabolism using the Oroboros Oxygraph 2k [7, 57] or the Seahorse XF24 analyser [19, 58]. In particular, Feeley et al. successfully measured the mitochondrial function of aortic sections. In the present work, in contrast to previous research [19], we applied 96-well Seahorse Spheroid Microplates to fit single 3D samples of aortic rings at the bottom of the wells, with one aortic ring per well, placed perpendicularly to the bottom of the plate (Fig. 1). An unquestionable advantage of this methodological approach was the possibility of evaluating energy metabolism parameters in a single 1-mm-long aortic ring, allowing comparison of different experimental conditions in aorta samples derived from the same mouse and with multiple technical replicates. Our approach was also proven to be suitable for analysis or vascular bioenergetics after ex vivo aorta incubation for up to 24 h, but the aorta should be incubated as longer sections and cut into rings before the assay for reliable measurements (data not shown). Another advantage of the optimised Seahorse-based methodology presented here was the good sensitivity and reproducibility of the results, achieved after normalisation of the functional results for protein levels. On the basis of our experience, the applied assay for the measurement of protein concentration in aortic rings represented the most accurate normalisation approach, as considering the minute size of aortic rings; it was troublesome to weigh them accurately. Furthermore, although we put considerable emphasis on preparing exceptionally even aortic rings for the assay, it was clearly seen that the thickness of the aortic wall was increased in aged mice, and hence the protein content was also higher. Therefore, normalisation of the functional readout for protein level was a prerequisite for reliable functional analysis of vascular energy metabolism in further studies using this methodology.

It needs to be underlined that despite optimalisation of the Seahorse-based methodology that may now provide robust and reproducible insight into the functional status of vascular metabolism, there are a couple of limitations of this approach that need to be addressed. Firstly, ex vivo samples of the aorta incubated in a culture medium or buffer under static conditions not exposed to flow conditions, even if supplemented with energetic substrates, may display to some extent an altered profile of basal vascular metabolism as compared to in vivo conditions. Secondly, we used classical substrates (glucose, pyruvate, and glutamine), but functional results could be different when a cocktail of substrates is used to better mimic in vivo situation (including, e.g. free fatty acids, amino acids). Thirdly, the results provide insight into the overall bioenergetics of the aortic wall, consisting of the single layer of endothelial cells, and VSMCs constituting the majority of the aortic ring mass. Therefore, it cannot be certain whether endothelial cells contribute to the observed changes and if so, to what extent. Finally, the analysis of glycolytic parameters with Seahorse is based on ECAR measurement proportional to extracellular lactate efflux; however, produced lactate can also be oxidised and used as an energy source, affecting the ECAR readout. CO_2_ produced in mitochondria can increase extracellular acidification as well. Therefore, it should be considered to support functional changes in glycolytic rate obtained with Seahorse by metabolomic analysis including targeted fluxomics. Having the insight into the fluxes of glycolysis as well as TCA and other pathways separately would further supplement the optimised Seahorse-based functional assay for mechanistic insight into altered vascular metabolism.

Taken together, on a methodological level, this work confirmed the robustness of the presented methodology for studying the functional profile of vascular energy metabolism in single rings of isolated murine aorta. Despite the limitations, this methodology may prove very useful in pharmacological studies aimed at finding novel pharmacotherapeutic approaches to reverse age-related changes in vascular phenotype.

## Conclusions

In summary, taking advantage of the optimised methodology for the functional analysis of vascular energy metabolism in isolated single rings of the murine aorta, we demonstrated that arterial stiffness and vascular remodelling in aged mice were associated with an impaired reserve of mitochondrial and glycolytic energy metabolism in aortic rings that could be attributed to vascular deficiency in NAD. Although vascular mitochondrial dysfunction has previously been reported to contribute to the vascular ageing phenotype [6, 36, 59] and NAD precursor prevented vascular ageing [34], in the present work, by studying functional responses in single rings of isolated murine aorta, we showed that the vascular ageing phenotype could be clearly linked to the impaired energy metabolism reserve of two major ATP-generating pathways in the vascular wall: mitochondrial respiration and glycolysis.

### Supplementary Information

Below is the link to the electronic supplementary material.

Supplementary file1 (PDF 384 KB)

## Data Availability

Data supporting the findings of this study are available from the corresponding authors upon reasonable request.

## Abbreviations

DMEM: Dulbecco’s modified Eagle medium
ECAR: Extracellular acidification rate
FBS: Foetal bovine serum
FCCP: Mitochondrial uncoupler (carbonyl cyanide 4-[trifluoromethoxy]phenylhydrazone)
GST: Glycolysis stress test
MST: Mitochondrial stress test
MEM: Eagle’s minimum essential medium
NAD: Nicotinamide adenine dinucleotide
NOS: Nitric oxide synthase
OCR: Oxygen consumption rate
PWV: Pulse wave velocity
R/A: Rotenone with antimycin A
VSMCs: Vascular smooth muscle cells
SILAC: Stable isotope labelling with amino acids in cell culture
UPLC-MS/MS: Ultra-performance liquid chromatography tandem mass spectrometry
