# Supplementary material for: Functional deterioration of vascular mitochondrial and glycolytic capacity in the aortic rings of aged mice
Source: GeroScience. 2024 Feb 28;46(4):3831–44. doi: 10.1007/s11357-024-01091-6 (PMC11226416; doi:10.1007/s11357-024-01091-6)
Supplement: Supplementary file 1 — Supplementary file1 (PDF 384 KB) [file 11357_2024_1091_MOESM1_ESM.pdf]

GeroScience – Supplementary information

Functional deterioration of vascular mitochondrial and glycolytic capacity in the aortic rings of aged mice

Agnieszka Karaś[1,2], Anna Bar[1], Kanchana Pandian[3], Agnieszka Jasztal[1], Zuzanna Kuryłowicz[1], Barbara Kutryb-Zając[4], Elżbieta Buczek[1,2], Stefano Rocchetti[1], Tasnim Mohaissen[1], Agata Jędrzejewska[4], Amy C Harms[3], Patrycja Kaczara[1], Stefan Chłopicki[1,5]

[1]Jagiellonian University, Jagiellonian Centre for Experimental Therapeutics, Bobrzynskiego 14, 30-348 Krakow, Poland

[2]Jagiellonian University, Doctoral School of Exact and Natural Sciences, Lojasiewicza 11, 30-348 Krakow, Poland

[3]Leiden University, Leiden Academic Centre for Drug Research, Einstein Road 55, 2333 CC Leiden, The Netherlands

[4]Medical University of Gdansk, Department of Biochemistry, Debniki 1, 80-211 Gdansk, Poland

[5]Jagiellonian University Medical College, Department of Pharmacology, Grzegorzecka 16, 31-531 Krakow, Poland

Corresponding authors:

Stefan Chlopicki, Jagiellonian Centre for Experimental Therapeutics, Jagiellonian University, Bobrzynskiego 14, 30-348 Krakow, Poland; tel: +48 12 6645464; fax: +48 12 2974615; email: stefan.chlopicki@jcet.eu

and

Patrycja Kaczara, Jagiellonian Centre for Experimental Therapeutics, Jagiellonian University, Bobrzynskiego 14, 30-348 Krakow, Poland; tel: +48 12 6645464; fax: +48 12 2974615; email: patrycja.kaczara@jcet.eu

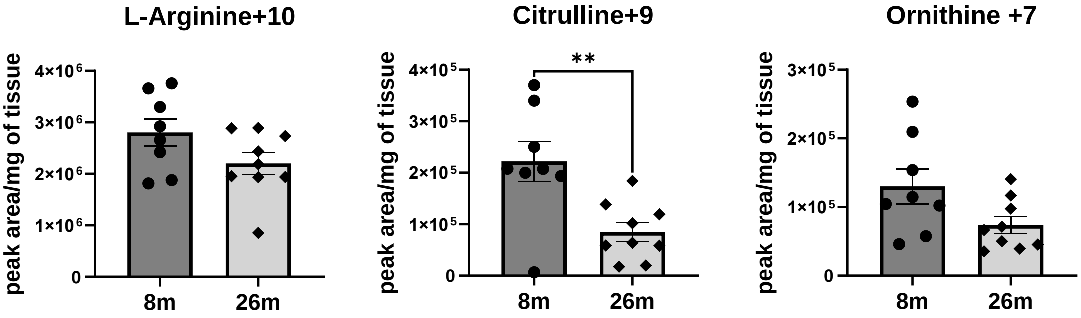

Figure S1. **Metabolism of stable isotope labelled L-Arginine in the aorta of young and old mice - metabolite levels.**

Aorta isolated from adult (8-month-old) and old (26-month-old) C57BL/6 mice (n=8-9) was incubated for 24h with $^{13}C_6$, $^{15}N_4$ L-Arginine-HCl and stimulated with calcium ionophore for the last 90 min. The levels of labelled arginine and its metabolites were measured using LC/MS method. The results were normalized for the wet tissue weight. Data represent means ±SEM, analysed with t-test, *p≤0.05.

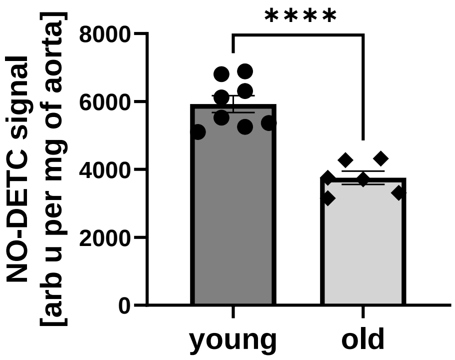

Figure S2. **Impairment of aortic NO production in the aorta of old C57BL/6 mice as compared with young C57BL/6 mice measured ex vivo with electron paramagnetic resonance (EPR).**

Stimulated nitric oxide production in the isolated abdominal aorta was evaluated in young (3-month-old) and old (24-month-old) C57BL/6 mice. NO was trapped with diethyldithiocarbamic acid sodium salt ($Fe^{2+}(DETC)_2$) and measured as NO-$Fe^{2+}(DETC)_2$ signal with electron paramagnetic resonance (EPR) spectroscopy using EMX Plus spectrometer (Bruker, Germany) as previously described [1,2]. The results were normalized for the wet tissue weight. Data represent the means ± SEM (n=6-8), analysed with t-test, ****p≤0.001.

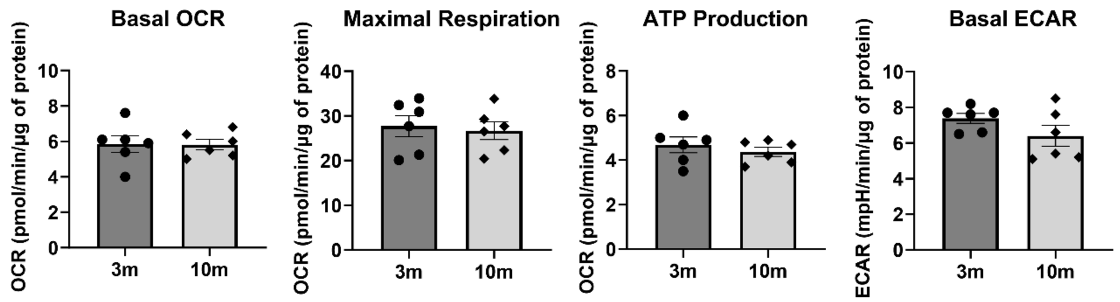

Figure S3. **Basal vascular energy metabolism functional profile in isolated rings of 10-month-old and 3-month-old C57BL/6 mice.**

Vascular metabolism in aortic rings was measured using the Seahorse XFe96, mitochondrial stress test. Data represent the means ± SEM (n=6), analysed with t-test.

References

[1] Przyborowski K, Proniewski B, Czarny J, Smeda M, Sitek B, Zakrzewska A, et al. Vascular Nitric Oxide–Superoxide Balance and Thrombus Formation after Acute Exercise. Medicine & Science in Sports & Exercise 2018;50:1405–12. https://doi.org/10.1249/MSS.0000000000001589.

[2] Bar A, Targosz-Korecka M, Suraj J, Proniewski B, Jasztal A, Marczyk B, et al. Degradation of Glycocalyx and Multiple Manifestations of Endothelial Dysfunction Coincide in the Early Phase of Endothelial Dysfunction Before Atherosclerotic Plaque Development in Apolipoprotein E/Low-Density Lipoprotein Receptor-Deficient Mice. Journal of the American Heart Association 2019;8. https://doi.org/10.1161/JAHA.118.011171.
